# The Aging Narcissus: Just a Myth? Narcissism Moderates the Age-Loneliness Relationship in Older Age

**DOI:** 10.3389/fpsyg.2018.01254

**Published:** 2018-07-24

**Authors:** Gregory L. Carter, Melanie D. Douglass

**Affiliations:** Department of Psychology, York St John University, York, United Kingdom

**Keywords:** narcissism, loneliness, age, mental health, adaptation

## Abstract

**Objective:** Recent research has indicated that sub-clinical narcissism may be related to positive outcomes in respect of mental and physical health, and is positively related to an extended lifespan. Research has also indicated narcissism levels may decline over the lifespan of an individual. The aims of the present study were to investigate these issues, exploring age-related differences in levels and outcomes of narcissism. Specifically, narcissism’s relationship with loneliness, a deleterious but pervasive state among older-age individuals, was assessed.

**Methods:** A total of 100 middle-aged (*M_AGE_* = 48.07; *SD* = 5.27; 53% female) and 100 older-aged participants (*M_AGE_* = 70.89; *SD* = 5.97; 51% female) completed the 40-item Narcissistic Personality Inventory and the UCLA Loneliness Scale, Version 3.

**Results:** Older-age participants had significantly lower levels of narcissism, and significantly higher levels of loneliness than middle-aged participants. Age and narcissism significantly predicted self-reported loneliness levels, with narcissism moderating the relationship between age and loneliness.

**Conclusion:** This study supports existing work, indicating that a degree of narcissism is of benefit to psychological functioning in respect of age-related loneliness, and is found to be a protective factor in mental health.

## Introduction

Sub-clinical narcissism is operationally defined as ‘self-admiration that is characterized by tendencies toward grandiose ideas, fantasied talents, exhibitionism, and defensiveness in response to criticism; interpersonal relationships… characterized by feelings of entitlement, exploitativeness, and a lack of empathy’ (Raskin and Terry, 1988, p. 896). Some psychologists have considered the trait to be maladaptive (Freud, 1914; Kernberg, 1975; Washburn et al., 2004), and there are undoubtedly ‘costs’ associated with the trait. These include difficulty in maintaining relationships (social and romantic) over time (Campbell et al., 2005; Rauthmann, 2012). However, evidence has linked narcissism to a number of ‘bright’ outcomes. Narcissism is related to positive impression formation (Back et al., 2010), and an outgoing social style (Holtzman et al., 2010). Other benefits are related to evolutionary ‘fitness’: increased levels of lifetime sexual partners (Holtzman and Strube, 2012), achieving status in hierarchical environments (Maccoby, 2000; Rosenthal and Pittinsky, 2006), and a longer lifespan with good emotional, psychological and social well-being, and good mental health (Jonason et al., 2015). The present study explores the relationship between narcissism and a specific, deleterious mental state – loneliness – experienced by many in older age, and related to depression and higher levels of suicide (Battegay and Mullejans, 1992; Heisel et al., 2007; Singh, 2015). Existing evidence suggests narcissism decreases with age (Foster et al., 2003); the opposite is true of loneliness (Routasalo et al., 2006). The relationship between the two, and whether narcissism’s evolutionarily beneficial effects extend to ‘protect’ against loneliness are explored in the present study.

Historically, approaches to conceptualizing and studying narcissism were largely psychodynamic or psychoanalytical (Freud, 1914; Kohut, 1966). Nevertheless, there was a lack of consensus as to a singular, definitive classification of the concept (see Consoling, 1999). Decades of clinical, observational work ultimately led to the inclusion of a definition of narcissism in the third edition of the Diagnostic and Statistics Manual as a lack of empathy accompanied by a pervasive pattern of grandiosity (in fantasy or behavior) and a need for admiration (American Psychiatric Association, 1980). Narcissism was later classified as a discrete disorder (a ‘Cluster B Personality Disorder’) in the DSM-IV, according to the criteria of possessing a grandiose sense of self-importance; a preoccupation with fantasies of unlimited success, power, brilliance, beauty, or ideal love; a belief that he or she is ‘special’ and ‘unique’; a requirement for excessive admiration; and a sense of entitlement. Over a similar period to these developments, Raskin and Hall (1979) created the Narcissistic Personality Inventory (NPI). Later refined by Raskin and Terry (1988), the NPI is considered the genesis of a different approach to the trait. The NPI provided the impetus to view narcissism from a quantitative, empirical, and social-personality point of view, and to define and explore a sub-clinical level of the character trait (Miller and Campbell, 2008).

Although associated with multiple adverse attitudes and behaviors, evolutionary psychologists have recently appraised narcissism in respect of the benefits that high levels of the trait confer, as well as its costs. Holtzman and Strube (2012) suggest the charm, self-adornment, and unrestricted sociosexuality associated with the trait facilitate a successful short-term mating strategy. Paunonen et al. (2006) found that the egotism and self-esteem aspects of narcissism were associated with other-rated leadership potential. Jonason et al. (2015) found that narcissism was related to living longer, and to feelings of hope, self-esteem and psychological well-being. Jonason et al. (2015) argued that this was an example of the adaptive benefits that narcissism yields; in particular that positive mental health outcomes were a result of narcissistic individuals’ extraversion and sociability. This was proposed to partly stem from narcissistic individuals’ need for others to admire and lavish attention on them.

### Narcissism and Age

Despite myriad empirical studies exploring the trait’s correlates and outcomes, little attention has focused on the relationship between narcissism and age. In particular, there is a paucity of work on narcissism involving older-age participants: most studies have been conducted with student- or early-middle-aged participants. A recent meta-analysis of 355 studies (*N =* 470,856) reported that the upper end of the age range of participants was 55 years old (Grijalva et al., 2015). Among the few authors who have reflected on narcissism in later life, most have adopted case-study approaches (Jovic, 1986; Becker, 2006). Even the Berlin Aging Study (BASE; Lindenburger et al., 2010), a large-scale study that investigated the mental health and psychological functioning of older-age participants (aged 70–100), did not address the issue, despite its focus on sub-diagnostic psychopathology, and self and personality concepts.

Where studies have been conducted on this topic, researchers have noted that increasing age can affect narcissistic tendencies, relating to typically contemporaneous changes to an individual’s role in the world (Wheelock, 1997). These changes may encompass a lessening of authority and responsibility, and even a loss of independence (Danko et al., 2009). Researchers have proposed that this creates ‘narcissistic injury’ (Wheelock, 1997) and induces a ‘narcissistic crisis’ (Teising, 2008). Changes in self-perception are particularly deleterious to narcissism, particularly regarding sentiments of self-admiration, fantasied talents, and exhibitionism. Societal views of the age group to which one belongs greatly affect the individuals (Danko et al., 2009). Battegay and Mullejans (1992) concluded that the elderly have ‘less narcissism at their disposal’ (p. 293); a view shared by Pellerin et al. (2003), who referred to ‘the weakening of narcissism’ (p. 89).

Supporting this, Twenge and Campbell (2008), in a large-scale (*N* = 3,445), geographically and ethnically wide-ranging cross-sectional study, reported that college-aged members of ‘Generation Me’ (p. 862) are more narcissistic than post-war ‘Baby Boomers’ and their antecedents. Although contested by some (Trzesniewski et al., 2008), a meta-analysis by Twenge et al. (2008) showed that NPI scores were significantly positively correlated with the year of their recording. Other available evidence (Roberts et al., 2010) suggests this is accurate, reflecting the recession of narcissism over age, and that age, rather than cohort belonging, *per se*, is the cause of this decline.

Extant research thus suggests narcissism decreases with age, simultaneous to a decrease in libido, self-attention, ego, and, often, socially conventional standards of beauty (Battegay and Mullejans, 1992). One of the consequences of the attenuation of narcissism may be the issues of self-confidence that arise within some elderly individuals. Older adults can experience self-confidence issues in interpersonal interactions, particularly with unfamiliar individuals. This extends to uncertainty in novel environments, or situations (Battegay and Mullejans, 1992). The effect, therefore, can be a greater feeling of isolation, and a tendency to withdraw from such novel people and circumstances, eliminating the social lifestyle that Jonason et al. (2015) hypothesized was central to narcissism’s health benefits. While Battegay and Mullejans’ (1992) Swiss study centers on the extreme outcomes these feelings can result in (i.e., suicide), their data are nonetheless valuable in gaining an insight into a rarely-studied aspect of the elderly experience. Suicide levels were significantly higher for men over 60 (*p* < 0.05), with sex differences increasing in those over 70 (*p* < 0.01), concurrent with decreased levels of narcissism (Battegay and Mullejans, 1992). The same is true of the findings of Clark (1993), who suggested that, in certain cases, suicidal tendencies were brought about by life-changes that relate to age, compounded by decreased levels of narcissism. Even in non-suicidal elderly individuals, a positive correlation between narcissism and depression has been noted. Importantly, both these relationships remain when controlling for cognitive functioning (Heisel et al., 2007). These results reflect the ‘mixed-benefits’ nature of the trait, but more importantly, the link between narcissism and mental health, which we continue to explore here.

### Narcissism and Loneliness

Narcissism can be conceived, partially, as an evolved protector against some negative psychological states, of which loneliness is one, and from which other negative psychological states subsequently stem. Wada (2000) has suggested a degree of narcissism may be crucial (and therefore beneficial) to individuals’ functioning, no less in old age than in earlier life. If narcissism declines to the extent existing literature suggests, that would partly explain a predisposition towards the ‘psychological vulnerability’ (p. 885) often manifest in later years. Wada (2000) suggests the degradation of narcissism levels in the elderly may, in part, be responsible for increased susceptibility to depression, which in turn ‘can induce various kinds of physical illness’ (p. 887). This is supported by Pellerin et al. (2003) and Stucke and Sporer (2002): the latter investigated the relationship between narcissism and negative mental states, in terms of responses to ego threats, and found lower levels of narcissism predicted negative emotionality.

In addition to representing a buffer against negative psychological states, the levels of self-esteem and self-confidence associated with narcissism (Zeigler-Hill et al., 2008) impart a considerable, specific defense against feelings of loneliness. Sedikides et al. (2004) conducted five studies into the relationship between narcissism and psychological health. The second of these found that narcissism was inversely related to both daily and dispositional loneliness. Furthermore, in a longitudinal study that considered clinical, anecdotal, and empirical evidence, Joiner et al. (2008) found that the relationship between levels of narcissism and psychological health appears to be linear, and unidirectional.

Moreover, Taylor et al. (2003) presented evidence that narcissism acts as a defense mechanism – a concomitant of good mental health – and that the self-esteem and self-enhancement associated with a degree of narcissism is positively correlated with developing good interpersonal relationships. Taylor et al.’s (2003) results support Battegay and Mullejans (1992), who recorded a concurrent reduction in narcissism and self-confidence in the elderly, particularly with regard to experiences involving others. Avoidance of social interactions likely results in increased feelings of isolation and loneliness, further exacerbated over time, in the manner of a feedback loop (see also Rainer and Martin, 2012). Finally, in a series of Finnish studies, Kalliopuska (2008) reported links between low levels of narcissism and high levels of shyness, leading to feelings of isolation. This further supports the relationship between social isolation and loneliness. Interpersonal skills, socialization patterns, and social support have also been implicated in an individual’s vulnerability to developing, as well as recovery from, depression (Coyne, 1976; Billings et al., 1983; Joiner et al., 1992; Bieling and Alden, 2001), in a similar feedback loop. Other studies have found comparable results, noting that depression – which, as established, is a greater risk to those with lower levels of narcissism – and other ‘hardships originating from aging’ (p. 223), were causally related to loneliness (Routasalo et al., 2006).

Evolutionary psychologists have proposed that individual differences in personality both ‘create’ and ‘solve’ problems (e.g., Buss, 1993). This argument has been made in respect of narcissism and short-term mating (Holtzman and Strube, 2012); evidence suggests it may be true of narcissism and loneliness. Through an aversion to several harmful health behaviors, whether or not the motivation is to maintain attractiveness, narcissism is related to a comparatively healthy lifestyle and increased longevity (Jonason et al., 2015; Hudek-Knežević et al., 2016). To the extent that older age engenders loneliness, narcissism may additionally function to ‘solve’ this costly outcome.

### The Present Study

As narcissism declines with age, and loneliness increases, the design of the present study measures both of these in a targeted sample of middle- and older-age participants. The selection of tests to be used in the current project was informed by the existing literature. The design required a measure of narcissism, a measure of loneliness, and a screening test to assess cognitive function in participants, to ensure that inventory items were understood. Samuel and Widiger (2008) compared five narcissism scales with a range of conceptualizations of sub-clinical narcissism, and found more empirical support for the NPI as a measure of sub-clinical narcissism than any other inventory, in accordance with the theories of Paulhus (2001). NPI items assess extroversion, dominance, independence, self-esteem, and self-importance (Corry et al., 2008). In addition, the NPI is considered the optimum extant measure for assessing ‘narcissism as a psychological construct in the sense of personality variable’ (Ritter and Lammers, 2007, p. 55). The UCLA Loneliness Scale, Version 3 (Russell, 1996) has demonstrated a high internal consistency (with alpha values ranging from 0.89 to 0.94), and strong test–retest reliability (*r* = 0.73, over a 12-month period) (Shaver and Brennan, 1991). It has also been used successfully with elderly populations (Cutrona et al., 1986). To screen for shortcomings in cognitive functions that may result from older age (Crook et al., 1986), the Mini-Cog Test was selected (Borson et al., 2000). This test was chosen as it is more sensitive to mild memory-related cognitive impairments than other screening tests (a sensitivity of 76%, higher than conventional neuropsychological batteries) and is quick to administer and score (Borson et al., 2003). The primary reason for its inclusion was to ensure that all participants, across age groups, had comparable cognitive functioning. This was judged important as assorted issues (including around focus and confusion) are known to increase in prevalence after the age of 65 (the mean age of participants in the older group was 70.89).

In all cases, selected measures were comparatively concise modifications of earlier versions. This minimization of length was considered important, so as not to subject participants to fatigue and jeopardize response accuracy.

In line with theories regarding age-based differences in narcissism, and existing literature on loneliness, it was anticipated that older-aged participants would report lower levels of narcissism and higher levels of loneliness than middle-aged participants. Age was expected to be a negative correlate and predictor of narcissism and a positive correlate and predictor of loneliness. Narcissism was expected to be a negative predictor of loneliness. Given evidence regarding narcissism’s protective benefits in respect of mental health, it was expected that narcissism would moderate the relationship between age and loneliness.

## Materials and Methods

### Participants

A total of 200 participants, 100 of whom were middle-aged (35–55 years old) and 100 of whom were older (65–85 years old) were recruited through opportunity sampling in the local community. Participant age ranged from 35 to 85; the mean age of the middle-aged group was 48.07 years (*SD* = 5.27); the mean age of the older-age group was 70.89 years (*SD* = 5.97). Participants aged 55–65 years were not targeted as this range is considered a non-distinct category between late middle age and early older age; the United Kingdom Census supports this assertion (Office for National Statistics, 2001). Furthermore, the full onset of older age is not typically considered to occur in the United Kingdom until the eligibility to retire, with benefits, which currently occurs for all the United Kingdom nationals by the age of 65. The middle-aged group comprised 47 male and 53 female participants; the older-aged group comprised 49 males and 51 female participants.

### Materials, Procedure, and Research Design

The Mini-Cog Test (Borson et al., 2000) requires participants to remember three simple words, complete a drawing of a clock set to a specific time (10.45), and then recall the words. Recall of fewer than two words and a drawing error indicates memory impairment. A positive screening would have resulted in participants’ responses being marked for destruction, however, no participants screened positively for memory impairment.

To measure narcissism, participants completed the 40-item NPI (Raskin and Terry, 1988). Participants indicate agreement with one of two paired items – for example, ‘I am essentially a modest person’ (non-narcissistic choice) or ‘Modesty does not become me’ (narcissistic choice). Scores are created based on the number of narcissistic choices made. Internal consistency was good (α = 0.89).

To measure feelings of loneliness, participants completed the 20-item UCLA Loneliness Scale, Version 3 (Russell, 1996). Items include ‘How often do you feel you lack companionship?’; participants indicate the frequency with which they feel such states on four-point semantic difference (‘never,’ ‘rarely,’ ‘sometimes,’ or ‘always’). Internal consistency was good (α = 0.89).

Due to the age of many participants, internet administration was not felt to be prudent. Particularly when working with older-aged participants, in-person administration has been recommended (Lindenburger et al., 2010), as has the use of traditional pen-and-paper methods (Foster et al., 2003). Participants were supplied with a letter of introduction, encompassing briefing information and a consent form. This information reminded participants of their right to decline participation, and to withdraw at any point, in which case any data would be destroyed. To enable withdrawal from the study, after completion of the questionnaire, participants were asked to create an anonymous code and provided with the experimenter’s contact details to request this. Questions were then invited, before informed consent was obtained. Following consent, the experimenter administered the Mini-Cog test. Participants were given printed versions of NPI and UCLA Loneliness Scale to complete. Written instructions were provided for each measure; no participants required additional explanation, or assistance with writing their answers. Participants completed their forms in private. In keeping with the nature of items comprising the NPI and UCLA Loneliness Scale, particular consideration was given to participants’ privacy. Participants detached consent forms, and placed them in a marked envelope. Following their completion of the test inventories, participants placed answer sheets in a separate envelope.

## Results

All participants passed the Mini-Cog test. Descriptive statistics for participants’ age, NPI, and UCLA Loneliness Scale scores are presented in **Table 1**.

**Table 1:** Age, Narcissistic Personality Inventory (NPI), and UCLA Loneliness Scale scores.

	Minimum		Maximum		Mean (*SD*)	
	Middle-age	Older-age	Middle-age	Older-age	Middle-age	Older-age
Age (years)	35	65	55	83	48.07 (5.27)	70.89 (5.97)
NPI score	6	0	30	23	15.92 (5.13)	10.09 (5.57)
UCLA score	24	28	54	68	39.80 (7.64)	46.20 (9.97)

To assess differences between samples, *t*-tests were conducted on NPI and UCLA Loneliness scores between groups. Older-aged participants reported significantly lower levels of narcissism [*t*(198) = 7.7, *p* < 0.001] and significantly higher levels of loneliness than middle-aged participants [*t*(198) = 5.09, *p* < 0.001]. For both groups, males reported significantly higher narcissism scores than females (middle-aged, *p* < 0.05; older-aged, *p* < 0.01), in keeping with the majority of previous studies (Grijalva et al., 2015).

In order to test whether narcissism moderated the relationship between age and loneliness, a hierarchical linear regression was performed, first testing for the predictive utility of age alone, then age with narcissism, followed by an age-by-narcissism interaction (Aiken et al., 1991; Pedhazur, 1997). Narcissism scores were centered before the interaction term was computed in order to control for multicollinearity of the interaction term, which otherwise would have exceeded a VIF value of 10 (O’Brien, 1987). Due to the significant difference in narcissism scores found between male and female participants, sex was entered in the first step. All of the data met conservative measures of normality.

**FIGURE 1:**
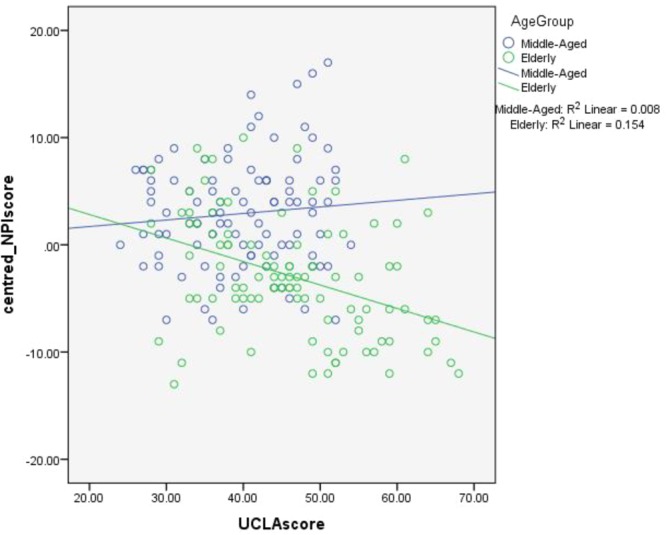
Interaction of age and narcissism in predicting loneliness ratings.

The hierarchical regression revealed that at stage one, age contributed significantly to the regression model, *F*(1, 198) = 25.947, *p* < 0.001 and accounted for 11.1% of the variance in loneliness scores. Introducing narcissism explained 14.0% of the variance in loneliness, which was significant, *F*(2, 197) = 17.149, *p* < 0.001. When the interaction term between age and narcissism was entered into the model, a total of 19.2% of the variance was explained, which was significant, *F*(3, 196) = 16.761, *p* < 0.001. Sex did not significantly contribute to the model at any stage and was therefore excluded from the analysis. The results suggest that narcissism moderates the relationship between age and loneliness; a graphical representation of the interaction can be found in **Figure 1**.

To explore the moderating effect of narcissism further, and to better understand the interaction between narcissism and age, correlation analyses of age and loneliness relationship were conducted. First, the full sample was tested. A significant positive correlation was found between age and loneliness *r*(198) = 0.35, *p* < 0.001. That is, older participants tended to have higher loneliness scores. Next, the correlation was re-tested, controlling for narcissism. It remained significant (positive) *r*(198) = 0.23, *p* < 0.001, but decreased in strength.

Finally, the correlation was tested within each participant group, controlling for narcissism in both cases. For the middle-aged group, the correlation was not significant *r*(98) = 0.02, *p* = 0.832. For the older-age group, the correlation was also not significant *r*(98) = 0.01, *p* = 0.947. Narcissism thus moderated the negative relationship between age and loneliness in both middle-aged and older-age participants. Within groups, results did not differ significantly by sex.

## Discussion

The results of the current study supported the hypotheses. Narcissism was lower in older-aged participants compared with middle-aged participants. Age was negatively correlated with, and negatively predicted narcissism, but was positively correlated with, and positively predicted loneliness. Narcissism was a negative correlate and predictor of loneliness. Narcissism also moderated the relationship between age and loneliness.

A lower level of narcissism in older individuals is in line with the conclusions of the limited number of studies that have previously explored this issue (Twenge and Campbell, 2008; Danko et al., 2009). The present study therefore reinforces existing evidence contradicting case studies that associate later life with increased narcissism (e.g., Peruchon, 2004). With regard to the potential effect of cohort (i.e., generational) membership (Trzesniewski et al., 2008; Roberts et al., 2010), correlation results bear scrutiny. Within 35–55 year olds (the middle-aged group), there was no significant relationship between narcissism and age, indicating no decline in narcissism across these years. However, 65 appears to be a threshold at which this relationship becomes significant. It therefore seems cohort membership is important, but only in so far as it reflects chronological age. This may be an effect of the substantial change in individuals’ lives and roles that typically occur after retirement (Wheelock, 1997; Danko et al., 2009). However, in respect of narcissism in relation to generational belonging, recent evidence from the BASE (Hülür et al., 2016) has indicated that the BASE II cohort, studied 2013–2014, reported significantly lower levels of loneliness than the 1990–1993 cohort. If Roberts et al.’s (2010) assertion is correct in that each generation is ‘more narcissistic than their elders’ (p. 97), this may be reflected in the findings of the BASE results (Hülür et al., 2016) and the current study. BASE does not presently record narcissism levels; this could be considered in the future.

A higher level of loneliness in older individuals is also in keeping with existing literature (Routasalo et al., 2006). In contrast to the negative relationship between age and narcissism, age, and loneliness are positively related in older-aged individuals. As with narcissism, this finding indicates that levels of loneliness remain relatively stable over middle-age, suggesting that loneliness is not a typical experience for this age group. Beyond 65, however, individuals are increasingly likely to feel lonely. This may be explained by the often decreased levels of socializing on the part of many older individuals (Kalliopuska, 2008), and the possible loss of close friends and even spouses (Teising, 2008). Higher levels of narcissism may provide one of several buffers against loneliness, in that individuals with higher levels of the trait are likely to continue to seek social interaction (Holtzman et al., 2010), even if only to seek attention.

The narcissism that significantly moderated the relationship between age and loneliness in older-aged participants supports previous research on the protective effects that narcissism can impart (Jonason et al., 2015; Hudek-Knežević et al., 2016), including in later life (Taylor et al., 2003; Zeigler-Hill et al., 2008). Higher levels of narcissism do impart a defense against certain negative mental states – implying that a degree of narcissism should indeed be seen as beneficial in this respect, and a contributing factor to good psychological health (Jonason et al., 2015). Additionally, narcissism is associated with multiple reasons to form friendships (Jonason and Schmitt, 2012), in turn increasing the likelihood of being able to draw on valuable social support (e.g., Cohen and Wills, 1985), offsetting loneliness, stress, and other negative mental states.

### Limitations and Future Research

While the current study does extend knowledge regarding narcissism’s relationship with age, it is cross-sectional. Longitudinal research would enhance comprehension of this relationship, especially in respect of the importance of age as compared with generational belonging. In addition, the present study depends on self-report data, which is another limitation. We hope the present study may act as a catalyst for additional work to be undertaken. Some preliminary work of a longitudinal nature has been undertaken with a younger sample (Carlson and Gjerde, 2009), but no such study has yet been presented including participants of middle- or older-age.

Future work could also consider other variables that may account for the association between narcissism and loneliness – such as self-esteem, extraversion, sociability, and neuroticism. While the NPI does tap these most of these traits (bar neuroticism; Miller and Campbell, 2008), inclusion of explicit measures to assess them may be enlightening. It is also important that the research investigate the importance of such factors in predicting loneliness in older age. Given the distinction between aloneness and loneliness, there are likely to be complex interactions of factors contributing to this relationship. This research represents only a first step in exploring this issue.

In addition, the measurement inventories selected for the current study’s questionnaire are not without fault. Miller and Campbell (2008) have suggested that there are problems within the current conceptualization of sub-clinical narcissism as measured by the NPI. Namely, these are that it primarily captures the antagonistic, conscientious, and extraverted components of the trait, but functions less well in capturing neurotic facets. Moreover, several items on Raskin and Terry’s (1988) NPI are explicitly future-oriented. These include ‘I will be a success,’ ‘I want to amount to something in the eyes of the world,’ and ‘I am going to be a great person.’ These may be inherently difficult for older-age individuals to endorse. The average lifespan in the United Kingdom is 77.2 years for males and 81.6 years for females (United Nations Department of Economics, 2007). Moreover, as these statements are in the future tense, these statements may cause a comparison between one’s present self and one’s future or ideal self. Research has shown that older adults perceive less discrepancy between their actual and ideal selves (Ryff, 1991). Moreover, in comparison with young and middle-aged adults, they perceive more stability in their personality across their past, present, and future selves (Ryff, 1991). This implies that they do not envisage changes in, for instance, ‘successfulness’ in the future, and therefore may prevent them from endorsing these statements, which make up 10% of the NPI. In addition, they may have already achieved major life goals, and may consider themselves to have reached peak success, which would further hinder their ability to endorse these statements. Therefore, assessing narcissistic tendencies in individuals of or over these ages with such times may be flawed.

Increasingly, psychometric research is utilizing item-response theory analyses, such as Mokken’s (1971) analysis, to assess personality tests as participants respond to them. Some research has already been undertaken on a short narcissism subscale of a popular measure that assesses the Dark Triad of narcissism, machiavellianism, and psychopathy (Paulhus and Williams, 2002; Carter et al., 2015). Similar scrutiny should be applied to the NPI, and other measures, especially where the study of individuals of substantially different ages is desirable. Items may need to be revised to ensure parity in item perception and response likelihood.

Relatedly, the independent yet overlapping traits comprising the Dark Triad share multiple correlates. Although they differ in respect of positive health- and mental-health-related outcomes (Jonason et al., 2015; Hudek-Knežević et al., 2016), measuring all three traits simultaneously increases confidence that conclusions regarding any of the traits are best attributed to that, and not another component of the Dark Triad (Furnham et al., 2014).

## Conclusion

The evidence from this study supports existing work indicating that a degree of narcissism supports normal psychological functioning. To this extent, narcissism appears to both ‘create’ and ‘solve’ at least some issues related to longevity. Increasing levels of narcissism in subsequent generations may be an on-going adaptive response to the longer lives that each generation, at least in many countries, can expect to have. The issue of changing levels of narcissism across the lifespan is undoubtedly complex; further research is needed to better understand the issue, and how it affects individuals in later life. Evidence has shown that narcissism is important to mental health, including loneliness, which has in turn been linked to other psychological issues, including an increased rate of suicide in the older-aged. Even in non-suicidal older-age individuals, a positive correlation between narcissism and depression has been noted. It is, therefore, extremely critical for the mental health of a large portion of the population that this issue is better-understood. In order to accurately assess narcissism in order age, revisions to existing inventories may be needed. This study increases the weight of evidence that recognizes the importance of narcissism as a protective factor in mental health and adds to calls for the relationship between age and narcissism to come under greater scrutiny – it is a long overdue reflection.

## Ethics Statement

This study was carried out in accordance with the recommendations of the ethical guidelines of the British Psychological Society and the University of Durham. Protocol was approved by the ethics committee of that University’s Psychology Department.

## Author Contributions

GC gathered the data and wrote the initial draft(s) of the manuscript. MD contributed to the additional analyses and proposed the amendments to the paper that improved its quality.

## Conflict of Interest Statement

The authors declare that the research was conducted in the absence of any commercial or financial relationships that could be construed as a potential conflict of interest.

## References

[B1] Aiken L. S. West S. G. Reno R. R. (1991). *Multiple Regression: Testing and Interpreting Interactions*. Thousand Oaks, CA: Sage.

[B2] American Psychiatric Association (1980). *Diagnostic and Statistical Manual of Mental Disorders* 3rd Edn. Washington, DC: American Psychiatric Association.

[B3] Back M. D. Schmukle S. C. Egloff B. (2010). Why are narcissists so charming at first sight? Decoding the narcissism–popularity link at zero acquaintance. *J. Pers. Soc. Psychol.* 98 132–145. 10.1037/a0016338 20053038

[B4] Battegay R. Mullejans R. (1992). Decreased narcissism in the aged and in suicide. *Swiss Arch. Neurol. Psychiatry* 143 293–306. 1279789

[B5] Becker D. (2006). Therapy for the middle-aged: the relevance of existential issues. *Am. J. Psychother.* 60 87–89. 10.1176/appi.psychotherapy.2006.60.1.87 16770918

[B6] Bieling P. J. Alden L. E. (2001). Sociotropy, autonomy, and the interpersonal model of depression: an integration. *Cogn. Ther. Res.* 25 167–184. 10.1023/A:1026491108540

[B7] Billings A. G. Cronkite R. C. Moos R. H. (1983). Societal-environmental factors in unipolar depression: comparisons of depressed patients and non-depressed controls. *J. Abnorm. Psychol.* 92 119–133. 10.1037/0021-843X.92.2.119 6863728

[B8] Borson S. Scanlan J. Brush M. Vitaliano P. Dokmak A. (2000). The mini-cog: a cognitive ‘vital signs’ measure for screening in multi-lingual elderly. *Int. J. Geriatr. Psychol.* 15 1021–1027. 10.1002/1099-1166(200011)15:11<1021::AID-GPS234>3.0.CO;2-6 11113982

[B9] Borson S. Scanlan J. M. Chen P. Ganguli M. (2003). The mini-cog as a screen for dementia: validation in a population-based sample. *J. Am. Geriatr. Soc.* 51 1451–1454. 10.1046/j.1532-5415.2003.51465.x 14511167

[B10] Buss D. M. (1993). Strategic individual differences: the role of personality in creating and solving adaptive problems. *Found. Pers.* 72 175–189. 10.1007/978-94-011-1660-2_13

[B11] Campbell W. K. Bush C. P. Brunell A. B. Shelton J. (2005). Understanding the social costs of narcissism: the case of the tragedy of the commons. *Pers. Soc. Psychol. Bull.* 31 1358–1368. 10.1177/0146167205274855 16143668

[B12] Carlson K. Gjerde P. (2009). Preschool personality antecedents of narcissism in adolescence and young adulthood: a 20-year longitudinal study. *J. Res. Pers.* 43 570–578. 10.1016/j.jrp.2009.03.003 20161614 PMC2811719

[B13] Carter G. L. Campbell A. C. Muncer S. Carter K. A. (2015). A Mokken analysis of the Dark Triad ‘Dirty Dozen’: sex and age differences in scale structures, and issues with individual items. *Pers. Individ. Dif.* 83 185–191. 10.1016/j.paid.2015.04.012

[B14] Clark D. C. (1993). Narcissistic crises of ageing and suicidal despair. *Suicide Life Threat. Behav.* 23 21–26. 8475529

[B15] Cohen S. Wills T. A. (1985). Stress, social support, and the buffering hypothesis. *Psychol. Bull.* 98 310–357. 10.1037/0033-2909.98.2.310 3901065

[B16] Consoling G. (1999). Kernberg versus Kohut: a (case) study in contrasts. *Clin. Soc. Work J.* 27 71–86. 10.1023/A:1022813515175

[B17] Corry N. Merritt R. D. Mrug S. Pamp B. (2008). The factor structure of the narcissistic personality inventory. *J. Pers. Assess.* 90 593–600. 10.1080/00223890802388590 18925501

[B18] Coyne J. C. (1976). Depression and the response of others. *J. Abnorm. Psychol.* 85 186–193. 10.1037/0021-843X.85.2.186 1254779

[B19] Crook T. Bartus R. T. Ferris S. H. Whitehouse P. Cohen G. D. Gershon S. (1986). Age-associated memory impairment: proposed diagnostic criteria and measures of clinical change—report of a national institute of mental health work group. *Dev. Neuropsychol.* 2 261–276. 10.1080/87565648609540348

[B20] Cutrona C. Russell D. Rose J. (1986). Social support and adaptation to stress by the elderly. *Psychol. Ageing* 1 47–54. 10.1037/0882-7974.1.1.47 3267379

[B21] Danko M. Arnaud C. Gely-Nargeot M. (2009). Identity and ageing: psychosocial approaches. *J. Psychol. Neuropsychiatry* 7 231–242. 10.1684/pnv.2009.0195 20031505

[B22] Foster J. D. Campbell W. K. Twenge J. M. (2003). Individual differences in narcissism: inflated self-views across the lifespan and around the world. *J. Res. Pers.* 37 469–486. 10.1016/S0092-6566(03)00026-6

[B23] Freud S. (1914). *On Narcissism: An Introduction*. Yale, CT: Yale University Press.

[B24] Furnham A. Richards S. Rangel L. Jones D. N. (2014). Measuring malevolence: quantitative issues surrounding the Dark Triad of personality. *Pers. Individ. Dif.* 67 114–121. 10.1016/j.paid.2014.02.001

[B25] Grijalva E. Newman D. A. Tay L. Donnellan M. B. Harms P. D. Robins R. W. (2015). Gender differences in narcissism: a meta-analytic review. *Psychol. Bull.* 141 261–310. 10.1037/a0038231 25546498

[B26] Heisel M. J. Links P. Conn D. van Reekum R. Flett G. (2007). Narcissistic personality and vulnerability to late-life suicidality. *Am. J. Geriatr. Psychiatry* 15 734–741. 10.1097/01.JGP.0000260853.63533.7d 17804827

[B27] Holtzman N. S. Strube M. J. (2012). “The intertwined evolution of narcissism and short-term mating,” in *The Handbook of Narcissism and Narcissistic Personality Disorder: Theoretical Approaches, Empirical Findings, and Treatments* eds Campbell W. K. Miller J. D. (Hoboken, NJ: John Wiley & Sons) 210–221. 10.1002/9781118093108.ch19

[B28] Holtzman N. S. Vazire S. Mehl M. R. (2010). Sounds like a narcissist: behavioral manifestations of narcissism in everyday life. *J. Res. Pers.* 44 478–484. 10.1016/j.jrp.2010.06.001 20711512 PMC2918908

[B29] Hudek-Knežević J. Kardum I. Mehiæ N. (2016). Dark triad traits and health outcomes: an exploratory study. *Psihol. Teme* 25 129–156.

[B30] Hülür G. Drewelies J. Eibich P. Düzel S. Demuth I. Ghisletta P. (2016). Cohort differences in psychosocial function over 20 years: current older adults feel less lonely and less dependent on external circumstances. *Gerontology* 62 354–361. 10.1159/000438991 26820135

[B31] Joiner T. Petty S. Perez M. Sachs-Ericsson N. Rudd M. (2008). Depressive symptoms induce paranoid symptoms in narcissistic personalities (but not narcissistic symptoms in paranoid personalities). *Psychiatry Res.* 159 237–244. 10.1016/j.psychres.2007.05.009 18423618

[B32] Joiner T. E. Alfano M. S. Metalsky G. I. (1992). When depression breeds contempt; reassurance seeking, self-esteem, and rejection of depressed college students by their roommates. *J. Abnorm. Psychol.* 101 165–173. 10.1037/0021-843X.101.1.165 1537962

[B33] Jonason P. K. Baughman H. M. Carter G. L. Parker P. (2015). Dorian Gray without his portrait: psychological, social, and physical health costs associated with the Dark Triad. *Pers. Individ. Dif.* 78 5–13. 10.1016/j.paid.2015.01.008

[B34] Jonason P. K. Schmitt D. P. (2012). What have you done for me lately? Friendship-selection in the shadow of the Dark Triad traits. *Evol. Psychol.* 10 400–421. 10.1177/147470491201000303 22947669 PMC10480828

[B35] Jovic N. (1986). Age, crisis and psychogeriatrics. *Swiss Arch. Neurol. Psychiatry* 137 293–306. 2433747

[B36] Kalliopuska M. (2008). Personality variables related to shyness. *Psychol. Rep.* 102 40–42. 10.2466/pr0.102.1.40-42 18481662

[B37] Kernberg O. (1975). *Borderline Conditions and Pathological Narcissism.* New York, NY: Jason Aronson.

[B38] Kohut H. (1966). Forms and transformations of narcissism. *J. Am. Psychoanal. Assoc.* 14 243–272. 10.1177/000306516601400201 5941052

[B39] Lindenburger U. Smith J. Mayer K. Baltes P. (eds) (2010). *The Berlin Aging Study* 3rd Edn. Berlin: Akademie Verlag.

[B40] Maccoby M. (2000). Narcissistic leaders: the incredible pros, the inevitable cons. *Harv. Bus. Rev.* 78 68–78.

[B41] Miller J. D. Campbell W. K. (2008). Comparing clinical and social-personality conceptualizations of narcissism. *J. Pers.* 76 449–476. 10.1111/j.1467-6494.2008.00492.x 18399956

[B42] Mokken R. J. (1971). *A theory and Procedure of Scale analysis.* The Hague: Mouton & Co 10.1515/9783110813203

[B43] O’Brien M. L. (1987). Examining the dimensionality of pathological narcissism: factor analysis and construct validity of the O’Brien Multiphasic Narcissism Inventory. *Psychol. Rep.* 61 499–510. 10.2466/pr0.1987.61.2.499 3432483

[B44] Office for National Statistics (2001). *Census.* London: ONS.

[B45] Paulhus D. (2001). Normal narcissism: two minimalist accounts. *Psychol. Inq.* 12 228–230. 10.1016/S0092-6566(02)00505-6

[B46] Paulhus D. L. Williams K. M. (2002). The dark triad of personality: narcissism, Machiavellianism, and psychopathy. *J. Res. Pers.* 36 556–563. 10.1016/j.leaqua.2006.06.003

[B47] Paunonen S. V. Lönnqvist J. E. Verkasalo M. Leikas S. Nissinen V. (2006). Narcissism and emergent leadership in military cadets. *Leadersh. Q.* 17 475–486.

[B48] Pedhazur E. J. (1997). *Multiple Regression in Behavioural Research* 3rd Edn. Belmont, CA: Wadsworth.

[B49] Pellerin J. Pinquier C. Potart C. (2003). Hysteria and ageing. *J. Psychol. Neuropsychiatry* 1 89–97. 15683945

[B50] Peruchon M. (2004). Identity and ageing: a metapsychological point of view. *J. Psychol. Neuropsychiatry* 2 125–131. 15683975

[B51] Rainer J. P. Martin J. (2012). *Isolated and Alone: Therapeutic Interventions for Loneliness*. Sarasota, FL: Professional Resource Press.

[B52] Raskin R. Hall C. S. (1979). A narcissistic personality inventory. *Psychol. Rep.* 45:590. 10.2466/pr0.1979.45.2.590 538183

[B53] Raskin R. Terry H. (1988). A principal component analysis of the Narcissistic Personality Inventory and further evidence of its construct-validity. *J. Pers. Soc. Psychol.* 54 890–902. 10.1037/0022-3514.54.5.890 3379585

[B54] Rauthmann J. F. (2012). The Dark Triad and interpersonal perception: similarities and differences in the social consequences of narcissism, Machiavellianism, and psychopathy. *Soc. Psychol. Pers. Sci.* 3 487–496. 10.1177/1948550611427608

[B55] Ritter K. Lammers C. (2007). Narcissism – Variable of personality and personality disorder. *Psychother. Psychom. Med. Psychol.* 57 53–60. 10.1055/s-2006-951922 17211773

[B56] Roberts B. Edmonds G. Grijalva E. (2010). It is developmental me, not generation me: developmental changes are more important than generational changes in Narcissism - commentary on Trzesniewski & Donnellan. *Perspect. Soc. Sci.* 5 97–102. 10.1177/1745691609357019 21243122 PMC3020091

[B57] Rosenthal S. A. Pittinsky T. L. (2006). Narcissistic leadership. *Leadersh. Q.* 17 617–633. 10.1016/j.leaqua.2006.10.005

[B58] Routasalo S. Savikko N. Tilvis R. Strandberg T. Pitkala K. (2006). Social contacts and their relationship to loneliness among aged people – A population-based study. *Gerontology* 52 181–187. 10.1159/000091828 16645299

[B59] Russell D. (1996). UCLA Loneliness Scale (Version 3): reliability, validity and factor structure. *J. Pers. Assess.* 66 20–40. 10.1207/s15327752jpa6601_2 8576833

[B60] Ryff C. D. (1991). Possible selves in adulthood and old age: a tale of shifting horizons. *Psychol. Aging* 6 286–295. 10.1037/0882-7974.6.2.286 1863398

[B61] Samuel D. Widiger T. (2008). Convergence of narcissism measures from the perspective of general personality functioning. *Assessment* 15 364–374. 10.1177/1073191108314278 18310592

[B62] Sedikides C. Rudich E. Gregg A. Kumashiro M. Rusbult C. (2004). Are normal narcissists psychologically healthy? Self-esteem matters. *J. Pers. Soc. Psychol.* 87 400–416. 10.1037/0022-3514.87.3.400 15382988

[B63] Shaver P. Brennan K. (1991). Measures of depression and loneliness. *Meas. Soc. Psychol. Attitud.* 1 196–209. 10.1016/B978-0-12-590241-0.50010-1

[B64] Singh S. D. (2015). Loneliness, depression and sociability in old age. *Int. J. Indian Psychol.* 2 73–79. 10.4103/0972-6748.57861 21234164 PMC3016701

[B65] Stucke T. Sporer S. (2002). When a grandiose self-image is threatened: narcissism and self-concept clarity as predictors of negative emotions and aggression following ego-threat. *J. Pers.* 70 509–532. 10.1111/1467-6494.05015 12095189

[B66] Taylor S. Lerner J. Sherman D. Sage R. McDowell N. (2003). Are self-enhancing cognitions associated with healthy or unhealthy biological profiles? *J. Pers. Soc. Psychol.* 85 605–615. 10.1037/0022-3514.85.4.605 14561115

[B67] Teising M. (2008). Narcissistic mortification of ageing men. *Int. J. Psychoanal.* 88 1329–1344. 10.1516/K060-0110-227H-1404 18055370

[B68] Trzesniewski K. Donnellan M. Robins R. (2008). Do today’s young people really think they are so extraordinary? - An examination of secular trends in narcissism and self-enhancement. *Psychol. Sci.* 19 181–188. 10.1111/j.1467-9280.2008.02065.x 18271867

[B69] Twenge J. M. Campbell S. M. (2008). Generational differences in psychological traits and their impact on the workplace. *J. Manag. Psychol.* 23 862–877. 10.1108/02683940810904367

[B70] Twenge J. M. Konrath S. Foster J. D. Campbell W. K. Bushman B. J. (2008). Egos inflating over time: a cross-temporal meta-analysis of the narcissistic personality inventory. *J. Pers.* 76 875–901. 10.1111/j.1467-6494.2008.00507.x 18507710

[B71] United Nations Department of Economics (2007). *World Population Prospects: The 2006 Revision* Vol. 261 New York, NY: United Nations Publications

[B72] Wada H. (2000). Problems and strategies in the treatment of mental disorders in elderly patients with physical illness. *Jpn. J. Geriatr.* 37 885–888. 10.3143/geriatrics.37.885 11193361

[B73] Washburn J. J. McMahon S. D. King C. A. Reinecke M. A. Silver C. (2004). Narcissistic features in young adolescents: relations to aggression and internalizing symptoms. *J. Youth Adolesc.* 33 247–260. 10.1023/B:JOYO.0000025323.94929.d9

[B74] Wheelock I. (1997). Psychodynamic psychotherapy with the older adult: challenges facing the patient and the therapist. *Am. J. Psychother.* 51 431–444. 10.1176/appi.psychotherapy.1997.51.3.431 9327109

[B75] Zeigler-Hill V. Clark C. B. Pickard J. D. (2008). Narcissistic subtypes and contingent self-esteem: do all narcissists base their self-esteem on the same domains? *J. Pers.* 76 753–774. 10.1111/j.1467-6494.2008.00503.x 18482357

